# AXL-Driven Stemness and Hedgehog Signaling in HER2-Positive Breast Cancer with Acquired Trastuzumab Resistance: Synergistic Potential of AXL and HER2 Co-Targeting

**DOI:** 10.3390/life16030371

**Published:** 2026-02-25

**Authors:** Asiye Busra Boz, Idris Er, Enric Arasanz Picher, Sneha Smarakan

**Affiliations:** 1 Department of Medical Biology, Faculty of Medicine, Recep Tayyip Erdogan University, Rize 53100, Turkey; 2 Department of Medical Biology, Faculty of Medicine, Karadeniz Technical University, Trabzon 53100, Turkey; 3 Institute of Cancer Therapeutics, School of Pharmacy, Optometry and Medical Sciences, Faculty of Health and Social Care, University of Bradford, Bradford BD7 1DP, UK

**Keywords:** bemcentinib, hedgehog, HER2-positive breast cancer, stemness trastuzumab-resistant

## Abstract

Stemness is a critical factor in tumor initiation, progression, metastasis, and resistance to treatment. The AXL receptor and hedgehog (Hh) signaling pathways play significant roles in regulating stemness, making them potential therapeutic targets. This study explores the involvement of AXL and hedgehog signaling in maintaining stemness and contributing to trastuzumab resistance in HER2-positive breast cancer. The expression of AXL and Hh markers was assessed in trastuzumab-resistant SKBR3 and HCC1954 cell lines and their parental counterparts. Trastuzumab resistance was associated with upregulation of AXL expression, with the GAS6/AXL axis identified as a regulator of stemness. Although inhibition of hedgehog signaling using GANT61 did not affect AXL expression, overexpression of AXL led to increased levels of hedgehog markers (e.g., *Gli1*, *Ptch1*) and stemness markers (e.g., *Sox2*, *Oct4*, *Nanog*), while silencing AXL resulted in their downregulation. Furthermore, AXL overexpression enhanced stemness in resistant cells, suggesting its role in resistance mechanisms. The combination of AXL inhibition and trastuzumab treatment significantly reduced stemness and hedgehog marker expression, indicating a synergistic effect. These results emphasize the pivotal role of AXL in regulating both stemness and hedgehog signaling in HER2-positive breast cancer. The study suggests that targeting both AXL and HER2 could be a promising strategy to overcome trastuzumab resistance and improve treatment outcomes.

## 1. Introduction

Cancer stem cells (CSCs), which retain stem-like properties such as self-renewal and differentiation, play a pivotal role in tumor initiation, growth, metastasis, and resistance to conventional therapies [1]. Two critical signaling pathways, AXL and hedgehog (Hh), have emerged as key regulators in the maintenance of CSCs, contributing to their survival and aggressiveness [2,3,4]. Dysregulation of these pathways not only enhances tumor aggressiveness but also fosters resistance to treatment, making them important targets for therapeutic intervention. AXL, a receptor tyrosine kinase, has been increasingly recognized for its role in sustaining CSC properties by promoting cell survival, proliferation, and invasion [5]. Its overexpression has been associated with various cancers and is linked to poor prognosis and resistance to chemotherapy and targeted therapies [5]. AXL is associated with increased expression of stemness markers like NANOG, OCT4, and SOX2 [6]. The overexpression of AXL correlates with aggressive tumor behavior, poor prognosis, and resistance to tyrosine kinase inhibitors (TKIs) and chemotherapy. Targeting AXL in cancer has emerged as a promising therapeutic strategy, particularly in overcoming drug resistance associated with CSCs. Several AXL inhibitors, such as bemcentinib (BGB324) and cabozantinib, are under clinical evaluation [7]. The inhibition of AXL signaling has been shown to reduce CSC populations, impair metastasis, and resensitize tumors to traditional therapies.

Similarly, the hedgehog signaling pathway, involved in stem cell regulation and tissue repair, is frequently dysregulated in cancers, driving CSC characteristics and contributing to therapy resistance [4,8] in several cancers, including breast, lung, and glioblastoma. Recent studies suggest that AXL and Hh pathways may work in concert to maintain CSCs and promote tumor aggressiveness [9]. Both pathways regulate processes such as epithelial-to-mesenchymal transition (EMT) and stem cell renewal, which are essential for tumor progression. Understanding the interplay between these two pathways offers new insights into CSC biology and potential strategies for overcoming therapy resistance.

This study investigates the interplay between AXL and hedgehog signaling in maintaining CSC characteristics, their contribution to tumor progression, and the therapeutic potential of co-targeting these pathways to enhance treatment efficacy. Specifically, the research focuses on HER-2-positive breast cancer models examining the regulatory role of AXL and hedgehog in CSCs. By elucidating the mechanistic crosstalk between these signaling networks, the study aims to identify novel therapeutic strategies targeting CSCs with the goal of improving patient outcomes with HER2-positive breast cancer.

## 2. Materials and Methods

### 2.1. Cell Culture and Development of Trastuzumab-Resistant Cells

The HER2-overexpressing breast cancer cell lines HCC1954 (ATCC CRL-2338) and SKBR3 (ATCC HTB-30) were selected for this study due to their documented ability to acquire resistance to trastuzumab. Cells were maintained in DMEM containing 10% fetal bovine serum, 1% sodium pyruvate, and 2 mM L-glutamine. To generate trastuzumab-resistant derivatives, parental HCC1954 and SKBR3 cells were continuously cultured in the presence of progressively increasing concentrations of trastuzumab, ranging from 0.1 to 10 μM, over approximately three months [10,11,12]. The acquisition of trastuzumab resistance was verified using an MTT-based cell viability assay by determining shifts in IC_50_ values. Prolonged exposure to trastuzumab resulted in a marked elevation of IC_50_ values, increasing from approximately 0.2 ± 0.01 to 2.6 ± 0.05 μM in SKBR3 cells and from 0.3 ± 0.01 to 2.4 ± 0.05 μM in HCC1954 cells. For transfection studies, HCC1954 and SKBR3 cells were seeded into six-well culture plates at a density of 2.2 × 10^5^ cells per well to obtain 40–60% confluence. Twenty-four hours after seeding, cells were transfected with 2 μg plasmid DNA per well using Lipofectamine 2000 (Waltham, MA, USA-Thermofisher) in accordance with the manufacturer’s instructions. Following transfection, cells were maintained in medium containing 1% FBS for 24 h and then stimulated with Gas6 (400 ng/mL; Abcam, ab131810, Cambridge, UK) for a further 24 h [13].

### 2.2. In Vitro Wound Healing Assay

Cells were plated to reach approximately 90% confluence and allowed to adhere overnight at 37 °C in a humidified atmosphere containing 5% CO_2_. Once a uniform monolayer was established, a linear wound was generated using a 200 µL pipette tip, and detached cells were removed by washing three times with PBS. Cells were then exposed to IC_50_ doses of bemcentinib alone, trastuzumab alone, or the combined treatment. Representative images of the scratched regions were acquired immediately after wounding (0 h) and following 72 h of incubation. The extent of wound closure was quantified using ImageJ software (version 1.53e; Java 1.8.0_172).

### 2.3. Real Time PCR

Total RNA was extracted from cell lysates using the RNeasy Kit (Qiagen, Venlo, The Netherlands) in accordance with the manufacturer’s guidelines. Reverse transcription was performed to generate complementary DNA (cDNA) using the iScript™ Reverse Transcription Supermix (Bio-Rad, Hercules, CA, USA). Quantitative real-time PCR analysis was conducted with the iTaq™ Universal SYBR^®^ Green One-Step Kit, and amplification was monitored on an Applied Biosystems ABI 7500 Real-Time PCR platform (Westminster, CO, USA) using 7500 Software v1. The thermal cycling conditions consisted of an initial denaturation at 95 °C for 10 s, followed by 40 amplification cycles of denaturation at 95 °C for 10 s, annealing at 60 °C for 1 min, and elongation at 72 °C for 30 s.

For the melting curve analysis, the protocol included an initial denaturation at 95 °C for 15 s, followed by an annealing phase at 60 °C for 1 min. The final step was performed at 95 °C for 15 s to complete the analysis.

Primers used for RT-qPCR were custom-designed using Primer3 software V3.1 and synthesized by Macrogen (Seoul, Republic of Korea) [11,14]. Each primer pair and sample was analyzed in triplicate to ensure technical accuracy, and all experiments were independently repeated three times using distinct biological samples to verify reproducibility. For RT-qPCR data analysis, Ct values of target genes were normalized to the housekeeping gene GAPDH. Normalized Ct values (ΔCt) were calculated as ΔCt = Ct (mean target gene) − Ct (mean housekeeping gene). Relative gene expression levels were determined using the 2^−ΔCt^ method, while fold changes were calculated using the 2^−ΔΔCt^ method, expressed as 2^−ΔCt^ (sample)/2^−ΔCt^ (control).

### 2.4. Combination Index (CI) Analysis

Drug–drug interactions were evaluated according to the Chou–Talalay approach using CompuSyn software v1.2 (ComboSyn Inc., Paramus, NJ, USA). Cells were exposed to increasing concentrations of trastuzumab and bemcentinib either as single agents or in fixed-ratio combinations. Concentration–response relationships were established using dose levels equivalent to 0.25×, 0.5×, 1×, 2×, and 4×, the respective IC_50_ values of each compound. The fraction affected (Fa) and combination index (CI) were subsequently determined, with CI values interpreted as synergistic (CI < 1), additive (CI = 1), or antagonistic (CI > 1) effects [15].

### 2.5. Western Blot

SKBR3 and HCC1954 cells were transfected with the AXL expression plasmid [16] (pFLAG AXL, Addgene plasmid #105933), which was generously provided by Rosa Marina Melillo (Addgene plasmid #105933; http://n2t.net/addgene:105933 (accessed on 12 December 2025); RRID: Addgene_105933), or with si-AXL (sc-29769, Santa Cruz, Dallas, TX, USA). To preserve integrin integrity, cells were collected by scraping instead of using trypsin-EDTA and subsequently lysed in TNTE buffer (Tris-NaCl-Triton X-100-EDTA) (Homemade, Tokyo, Japan). Protein samples were loaded onto a 7.5% acrylamide gel in running buffer containing 10% SDS, with Precision Plus Protein™ Dual Color Standards (Bio-Rad, Hercules, CA, USA) used as a molecular weight marker. Proteins were then transferred onto a PVDF membrane pre-soaked in methanol using a cold 1× transfer buffer on ice. Following transfer, the PVDF membranes were blocked in 5% skimmed milk prepared in TBST. After blocking, the membranes were incubated overnight at 4 °C with primary antibodies, including mouse monoclonal anti-AXL (sc-166269, Santa Cruz, Dallas, TX, USA) and mouse anti-α-tubulin (Sigma, Singapore), both diluted at 1:1000 in 5% skimmed milk in TBST. The following day, the membranes were incubated for one hour with the secondary antibody, goat anti-mouse IgG-HRP (Santa Cruz, Dallas, TX, USA), and diluted at 1:7500 in 5% skimmed milk in TBST. Protein bands were detected using Clarity™ Western ECL Substrate (Bio-Rad, Hercules, CA, USA) and visualized with the Bio-Rad ChemiDoc MP system. Data were normalized to α-Tubulin expression.

### 2.6. Colony Formation Assay

Plating efficiency for the colony formation assay was first evaluated, and a seeding density of 500 cells per well in 6-well plates yielded the highest efficiency; therefore, this density was used in all subsequent experiments. Drugs were applied at their respective IC_50_ concentrations, either as single agents or in combination, with DMSO serving as the control. Treated cells were incubated for 11 days in both fibrinogen-coated and uncoated 6-well plates. Following incubation, cells were washed with PBS, fixed in methanol, and stained with 0.1% crystal violet. Colonies were counted manually, and the surviving fraction was calculated using the following formula:

Surviving fraction (%) = (Number of colonies formed after treatment/Number of cells seeded) × 100

### 2.7. Statistical Analysis

Statistical evaluations were carried out using GraphPad Prism software v3. Comparisons among experimental groups were performed using two-way analysis of variance (ANOVA), with Tukey’s multiple comparison test applied as a post hoc analysis. A *p* value of ≤0.05 was used to define statistical significance. All data are expressed as mean ± standard deviation (SD) derived from three independent experiments, each performed in triplicate.

## 3. Results

### 3.1. AXL Expression Is Elevated in Trastuzumab-Resistant Cells and Is Not Regulated by Hedgehog Signaling

**Figure 1:**
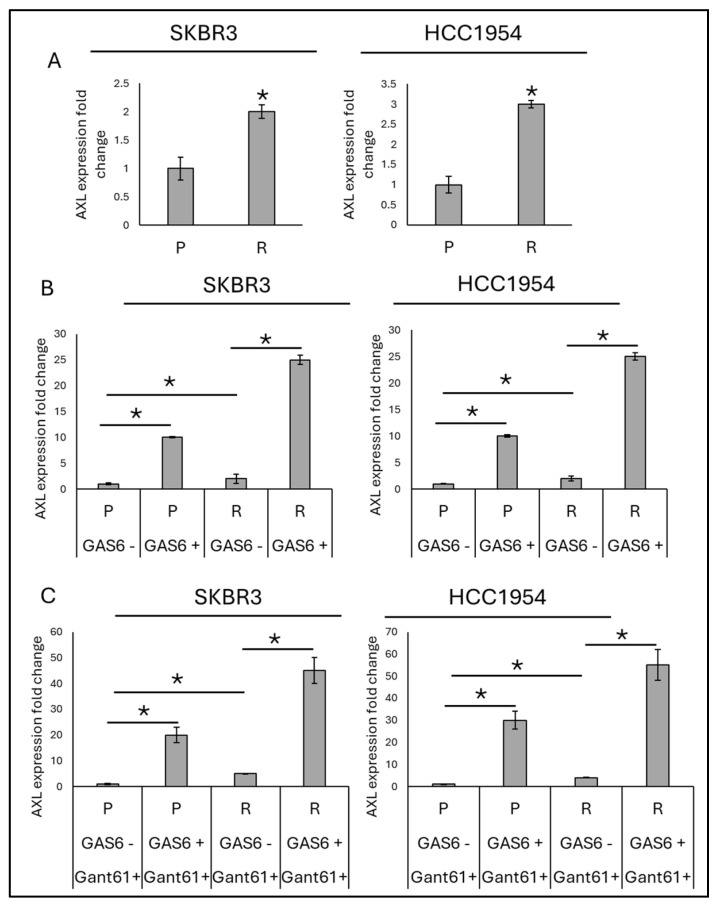
Expression of AXL in parental and trastuzumab-resistant breast cancer cells. (**A**) Differential expression of AXL expression in trastuzumab-resistant SKBR3 and HCC1954 cells as determined by qPCR. (**B**) in the presence or absence of the ligand, GAS6, (**C**) in the presence and absence of hedgehog inhibitor, GANT61 and GAS6. Two-way ANOVA followed by Tukey’s multiple comparisons post hoc test was used to determine statistical significance. Parental (P) and resistant (R) cell lines are indicated. Data are presented as mean ± SD (*n* = 3). * *p* ≤ 0.05.

To investigate the association of AXL expression with trastuzumab resistance, we analyzed AXL gene expression in trastuzumab-resistant SKBR3 and HCC1954 cell lines. The results revealed that AXL expression was significantly upregulated in these resistant cell lines compared to their parental counterparts. GAS6, a known ligand of AXL, was further used as a positive regulator for the AXL pathway. The presence of GAS6 was found to increase AXL gene levels in both parental and trastuzumab-resistant SKBR3 and HCC1954 cell lines (Figure 1).

To explore whether hedgehog signaling is associated with the GAS6/AXL axis, GANT61, an inhibitor of the downstream effector of the hedgehog pathway, *Gli1*, was added to SKBR3 and HCC1954 parental and resistant cells, both in the presence and absence of GAS6. Notably, GANT61 treatment did not impact the GAS6-mediated regulation of AXL in either parental or resistant cells. These findings suggest that the hedgehog pathway does not regulate the expression of AXL or participate in the GAS6/AXL axis in SKBR3 and HCC1954 cells, irrespective of their resistance to trastuzumab.

### 3.2. AXL Regulates Hedgehog-Responsive Gene Expression and Stemness

**Figure 2:**
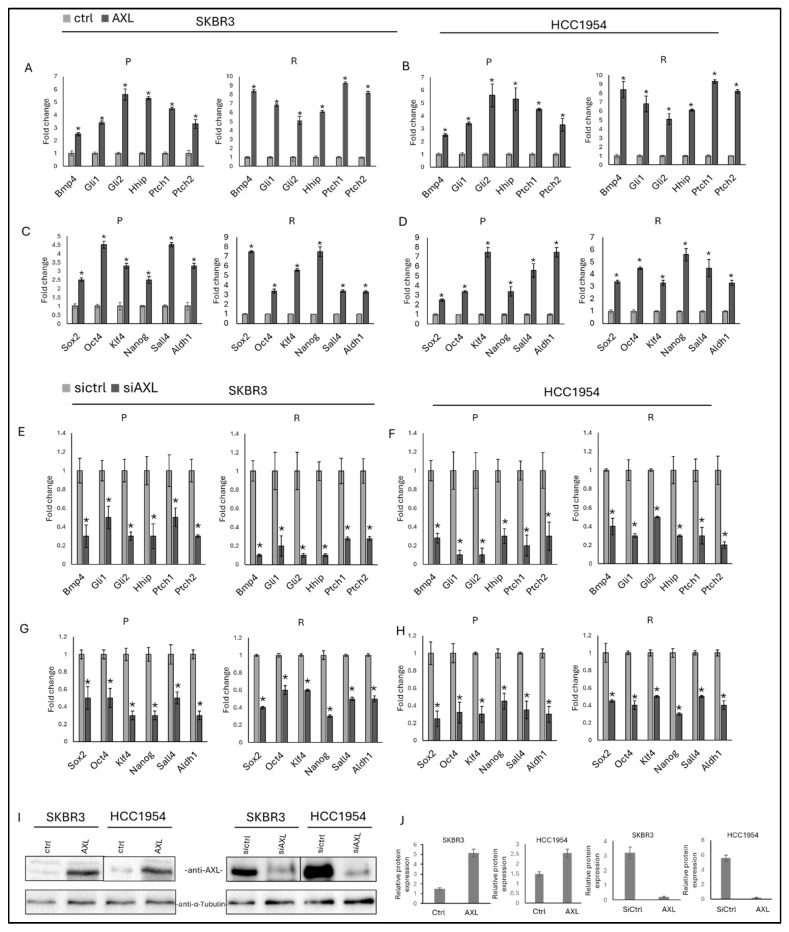
Overexpression of AXL increases the expression of hedgehog and stemness markers in SKBR3 and HCC1954 parental and resistant cells. Hedgehog-responsive gene expression in the presence of AXL overexpression (**A**) in SKBR3 parental and resistant and (**B**) HCC1954 parental and resistant cells. Stemness-responsive gene expression in the presence of AXL overexpression (**C**) in SKBR3 parental and resistant and (**D**) HCC1954 parental and resistant cells. Hedgehog-responsive gene expressions in presence and absence of siAXL (**E**) in SKBR3 parental and resistant cells and (**F**) in HCC1954 parental and resistant cells. Stemness-responsive gene expression in the presence and absence of siAXL, (**G**) in SKBR3 parental and resistant cells and (**H**) HCC1954 parental and resistant cells. (**I**) Representative Western blot showing AXL overexpression and silencing in SKBR3 and HCC1954 cells. (**J**) Quantification of band intensity corresponding to AXL overexpression and silencing in SKBR3 and HCC1954 cells. Two-way ANOVA followed by Tukey’s multiple comparisons post hoc test was used to determine statistical significance. Parental (P) and resistant (R) cell lines are indicated. Data are presented as mean ± SD (n = 3). * *p* ≤ 0.05.

To assess whether AXL influences hedgehog pathway signaling, AXL expression was either ectopically increased or suppressed in both parental and trastuzumab-resistant SKBR3 and HCC1954 cell lines, followed by evaluation of canonical hedgehog target genes, including *Bmp4*, *Gli1*, *Gli2*, *Hhip*, *Ptch1*, and *Ptch2*. Enforced AXL expression resulted in a pronounced induction of hedgehog-responsive gene expression (Figure 2), whereas depletion of AXL led to a significant reduction in the expression of these targets (Figure 2).

In parallel, the contribution of AXL to the regulation of stemness was investigated by examining the expression of key stemness-associated markers (*Sox2*, *Oct4*, *Klf4*, *Nanog*, *Sall4*, and *Aldh1*) under conditions of AXL overexpression (Figure 2C,D) or knockdown (Figure 2G,H). Elevated AXL levels consistently enhanced the expression of these stemness markers, while AXL silencing produced the opposite effect in both parental and resistant cell populations.

Collectively, these data demonstrate that AXL acts as a positive regulator of hedgehog signaling and stemness-associated gene expression in SKBR3 and HCC1954 cells, irrespective of trastuzumab sensitivity. Successful modulation of AXL expression was validated at the protein level, confirming the robustness of the experimental approach (Figure 2I,J).

### 3.3. AXL Expression Regulates Stemness Independent of GAS6 in HER2-Positive Breast Cancer Cells

**Figure 3:**
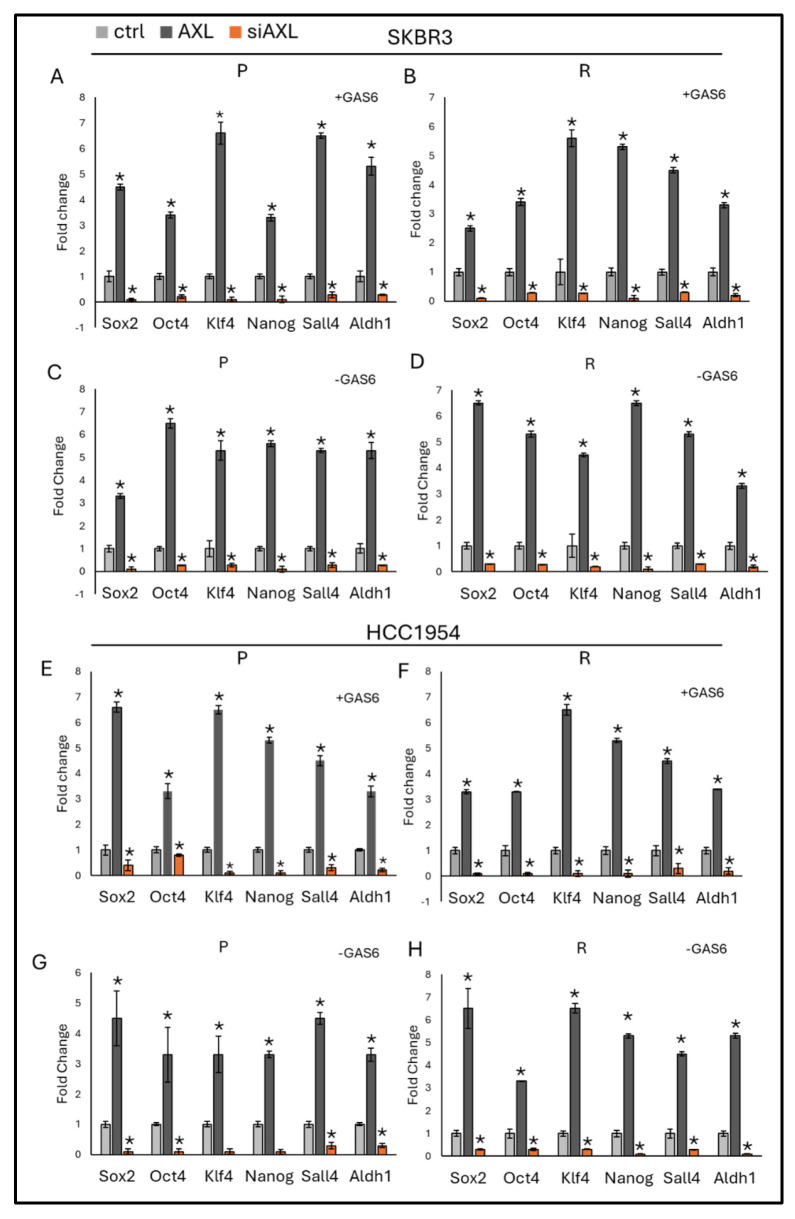
AXL expression regulates stemness independently of GAS6 in both parental and resistant SKBR3 and HCC1954 cell lines. Stemness-related gene expression: (**A**,**B**) In the presence of GAS6 in SKBR3 parental and resistant cells. (**C**,**D**) In the absence of GAS6 in SKBR3 parental and resistant cells. (**E**,**F**) In the presence of GAS6 in HCC1954 parental and resistant cells. (**G**,**H**) In the absence of GAS6 in HCC1954 parental and resistant cells. Two-way ANOVA followed by Tukey’s multiple comparisons post hoc test was used to determine statistical significance. Parental (P) and resistant (R) cell lines are indicated. Data are presented as mean ± SD (n = 3). * *p* ≤ 0.05.

To investigate the role of the GAS6/AXL axis in stemness regulation, the expression of stemness markers was analyzed under conditions of AXL overexpression or silencing, both in the presence and absence of GAS6. The results demonstrated that stemness marker expression significantly increased with AXL overexpression and decreased with AXL silencing in SKBR3 parental and resistant cells (Figure 3A–D) and in HCC1954 parental and resistant cells (Figure 3E–H), regardless of the presence of GAS6. This suggests that AXL regulates stemness independently of its ligand, GAS6 (Figure 3).

### 3.4. Dual Targeting of AXL and HER2 Induces Synergistic Effects in HER2-Positive Breast Cancer Cells

**Figure 4:**
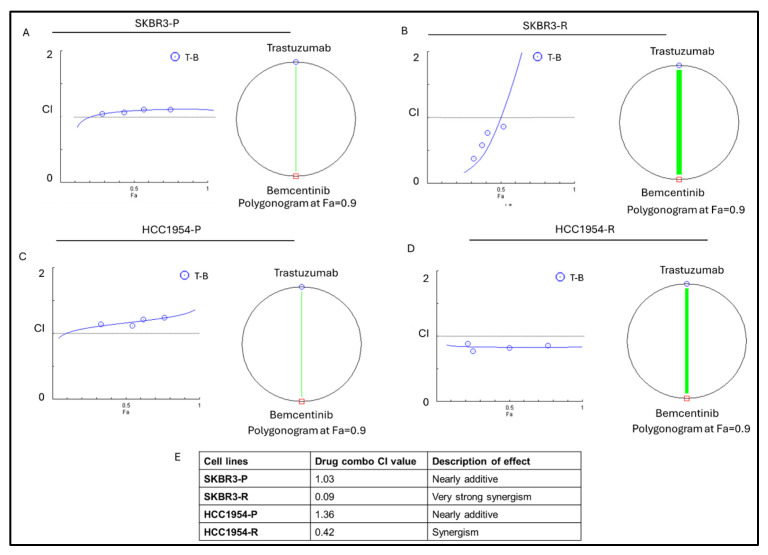
Trastuzumab–bemcentinib combination has a synergistic effect in SKBR3 and HCC1954 resistant cell lines. The graph of nearly additive effect in (**A**) SKBR3 and (**C**) HCC1954 parental cells. Very strong synergism and synergism in (**B**) SKBR3 and (**D**) HCC1954 resistant cells. (**E**) Combination index values of trastuzumab–bemcentinib drug combination. Parental (P) and resistant (R) cell lines are indicated. n = 3 ± SD.

The observed overexpression of the AXL pathway in HER2-expressing tumors prompted further investigation into the therapeutic potential of targeting this pathway to sensitize trastuzumab-resistant cells. Accordingly, Chou–Talalay combination assays were performed to determine and quantify drug–drug interactions. To evaluate the combined effects of trastuzumab and bemcentinib, cells were treated with five concentration points at a fixed 1:1 ratio for each agent: 0.25-fold, 0.5-fold, 1-fold, 2-fold, and 4-fold of the respective IC_50_ values of the individual drugs. Combination effects were assessed by calculating the combination index (CI) based on the median-effect principle described by Chou and Talalay [15,17]. Notably, the trastuzumab–bemcentinib combination exhibited a nearly additive effect in SKBR3-P and HCC1954-P cell lines, strong synergism in SKBR3-R cells, and synergism in the HCC1954-R cell line (Figure 4E).

**Figure 5:**
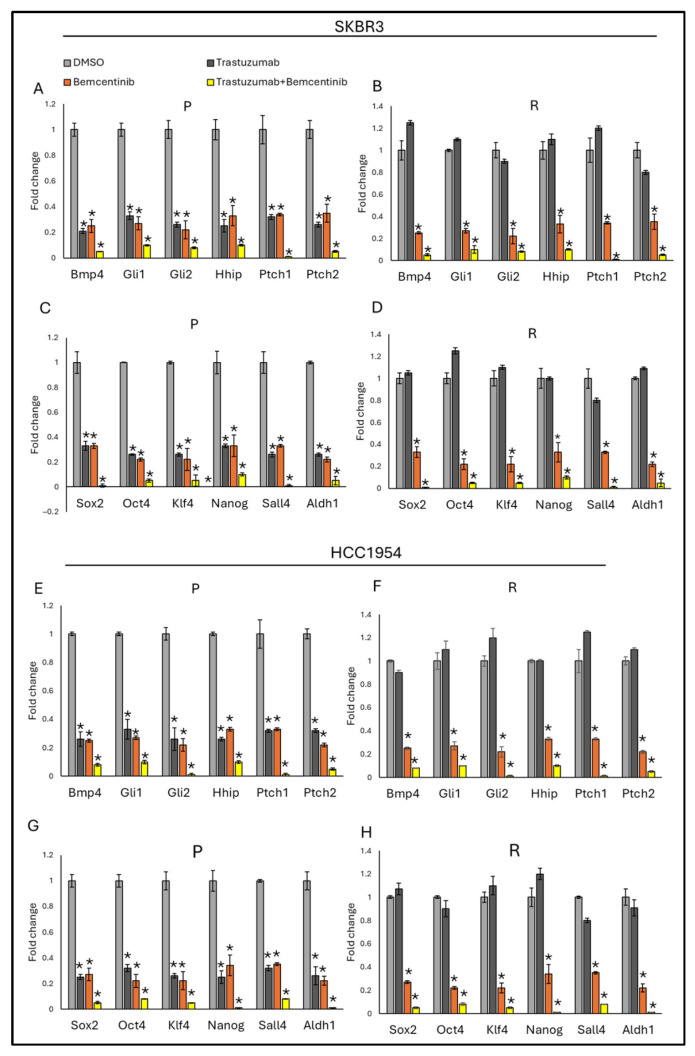
Trastuzumab–bemcentinib combination significantly downregulates hedgehog and stemness genes in SKBR3 parental and resistant cell lines. Hedgehog pathway-responsive gene expressions in the presence of trastuzumab, bemcentinib monotherapy, and combination therapy (**A**) in SKBR3 parental cells and (**B**) resistant cells. Stemness-responsive gene expressions in the presence of trastuzumab, bemcentinib monotherapy, and combination therapy (**C**) in SKBR3 parental cells and (**D**) in resistant cells. Hedgehog pathway-responsive gene expressions in the presence of trastuzumab, bemcentinib monotherapy, and combination therapy (**E**) in SKBR3 parental cells and (**F**) in resistant cells. Stemness-responsive gene expressions in the presence of trastuzumab, bemcentinib monotherapy, and combination therapy (**C**) in SKBR3 parental cells, (**D**) in resistant cells, (**G**) in HCC1954 parental cells, and (**H**) in resistant cells. Two-way ANOVA followed by Tukey’s multiple comparisons post hoc test was used to determine statistical significance. Parental (P) and resistant (R) cell lines are indicated. Data are presented as mean ± SD (n = 3). * *p* ≤ 0.05.

In SKBR3 parental cells, both bemcentinib and trastuzumab monotherapies reduced the expression of hedgehog-responsive genes (Figure 5A,B). However, no significant difference was observed between the effects of bemcentinib and trastuzumab groups on stem cell gene expression. In contrast, the combination treatment significantly decreased hedgehog-responsive gene expression compared to either monotherapy alone. Similar results were observed in HCC1954 cells (Figure 5E,F). In resistant SKBR3 and HCC1954 cells, trastuzumab monotherapy failed to reduce the expression of hedgehog-responsive and stemness-associated genes. However, in parental cells, trastuzumab monotherapy successfully reduced the expression of these genes. Bemcentinib monotherapy, on the other hand, decreased hedgehog-responsive and stemness-associated gene expression in both parental and resistant SKBR3 and HCC1954 cells, although there was no significant difference when compared to trastuzumab monotherapy (Figure 5A–D). Notably, the combination of trastuzumab and bemcentinib significantly downregulated both hedgehog-responsive and stemness-associated gene expression compared to either monotherapy in SKBR3 and HCC1954 parental and resistant cells. These findings highlight the potential synergistic effect of combining AXL inhibition with HER2-targeted therapy in HER2-positive breast cancer cells, particularly in overcoming resistance and downregulating stemness.

### 3.5. Bemcentinib and Trastuzumab Combination Therapy Reduces Migratory and Tumorigenic Properties

**Figure 6:**
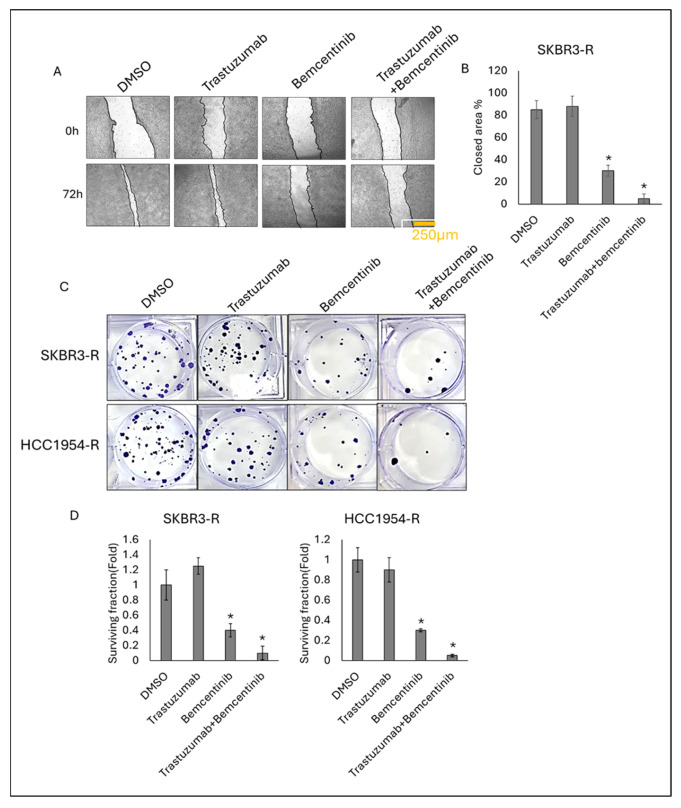
Combination therapy reduces migration and colony formation in resistant cell lines. (**A**) Representative images illustrating the effects of IC50 concentrations of trastuzumab, bemcentinib monotherapy, and their combination on wound closure in SKBR3-R cells. (**B**) Quantification of wound closure in SKBR3-R cells. (**C**) Representative images depicting the effects of IC_50_ concentrations of trastuzumab, bemcentinib monotherapy, and their combination on colony formation in SKBR3-R and HCC1954-R cells. (**D**) Quantification of colony formation in SKBR3-R and HCC1954-R cells. Two-way ANOVA followed by Tukey’s multiple comparisons post hoc test was used to determine statistical significance. Parental (P) and resistant (R) cell lines are indicated. Data are presented as mean ± SD (n = 3). * *p* ≤ 0.05. Scale bar: 250 µM.

To assess the functional impact of targeting AXL and HER2 in breast cells on cell migration, invasion, and proliferation, a wound healing assay was performed on SKBR3-R cells over a 72 h period. The results revealed that trastuzumab monotherapy did not affect wound closure in SKBR3-R cells due to their resistance (Figure 6A,B). However, bemcentinib monotherapy and its combination with trastuzumab significantly inhibited wound healing, indicating reduced migratory characteristics.

Further colony formation assays were conducted to evaluate the tumorigenic potential, survival, and tumor-initiating ability of SKBR3-R and HCC1954-R cells (Figure 6C,D). Cells were treated with bemcentinib, trastuzumab, and their combination for 11 days, and colonies were visualized and quantified using crystal violet staining. The results demonstrated that bemcentinib monotherapy significantly decreased colony formation compared to trastuzumab monotherapy in both SKBR3-R and HCC1954-R cells. Notably, the combination of bemcentinib and trastuzumab further reduced colony formation compared to either monotherapy (Figure 6C,D). These findings suggested that bemcentinib, particularly in combination with trastuzumab, effectively suppresses the migratory and tumorigenic properties of HER2-positive trastuzumab-resistant breast cancer cells, providing a promising therapeutic strategy.

## 4. Discussion

This study highlights the critical role of AXL signaling in regulating cancer stem cell (CSC) maintenance and drug resistance in HER2-positive breast cancer, potentially via modulation of the hedgehog pathway. Our findings provide new insights into the molecular crosstalk between these pathways and underscore the potential of dual therapeutic targeting to overcome trastuzumab resistance.

We confirmed that AXL expression is significantly elevated in trastuzumab-resistant SKBR3 and HCC1954 cell lines compared to their parental counterparts. Moreover, the AXL ligand GAS6 further upregulated AXL expression in both parental and resistant cells. These results align with previous studies implicating the GAS6/AXL axis in tumor progression, metastasis, and resistance to targeted therapies, including HER2 inhibition [18,19]. Importantly, inhibition of the hedgehog pathway with the *Gli1* antagonist GANT61 did not affect GAS6-induced AXL expression, indicating that hedgehog signaling does not regulate AXL, either directly or indirectly.

In contrast, we discovered that AXL positively regulates hedgehog signaling as AXL overexpression led to a significant upregulation of hedgehog-responsive genes (*Gli1*, *Gli2*, *Ptch1/2*, *Bmp4*), while AXL knockdown had the opposite effect. These findings suggest a unidirectional interaction where AXL acts upstream of hedgehog signaling in HER2-positive breast cancer cells. This shows that AXL contributes to maintaining oncogenic signaling, even in the presence of HER2 inhibition.

AXL expression also strongly correlated with the upregulation of stemness-associated transcription factors such as *Sox2*, *Oct4*, *Nanog*, *Klf4*, and *Aldh1*. Notably, this effect occurred independently of GAS6 stimulation, suggesting that AXL’s role in sustaining a CSC-like phenotype may be ligand-independent. As CSCs are known to drive tumor recurrence, metastasis, and therapy resistance [20], this finding identifies AXL as a key regulator of stemness in trastuzumab-resistant HER2-positive breast cancer.

Our data also reinforce the role of hedgehog signaling in maintaining CSC-like properties, consistent with previous literature [21,22]. Aberrant activation of this pathway enhances CSC gene expression and contributes to resistance against standard therapies. We propose that crosstalk between AXL and hedgehog pathways amplifies CSC-related phenotypes, posing a significant challenge for therapeutic intervention. As such, targeting one pathway in isolation may be insufficient due to compensatory activation of the other, allowing CSCs to survive and persist.

One of the most impactful findings of our study is that the dual inhibition of AXL and HER2 using bemcentinib and trastuzumab resulted in a synergistic antitumor effect, particularly in trastuzumab-resistant SKBR3 and HCC1954 cells. This combination more effectively suppressed hedgehog and stemness markers compared to single agents, with the Chou–Talalay analysis confirming strong to very strong synergy, especially in resistant populations. These results support the clinical potential of AXL/HER2 co-targeting to overcome resistance and reduce CSC-driven tumor progression.

Our findings support a model in which AXL drives resistance to HER2-targeted therapies by promoting hedgehog signaling and stemness—independent of GAS6. This makes AXL an appealing therapeutic target, particularly in the context of acquired resistance. Future work should validate these results *in vivo* and investigate the long-term effects of dual AXL/HER2 inhibition on recurrence, metastasis, and overall survival.

Additionally, this study raises important questions about the role of the tumor microenvironment (TME) and CSC plasticity in resistance. The interaction between CSCs, signaling networks, and the TME—through extracellular vesicles, cytokines, and stromal factors—likely contributes to the complexity of therapeutic resistance. Future studies should examine how the TME modulates AXL and hedgehog activity and whether targeting TME-associated factors may enhance the efficacy of AXL/HER2 dual inhibition.

## 5. Conclusions

In conclusion, this study provides compelling evidence that AXL and HER2 signaling pathways synergize to sustain CSC properties and confer resistance to trastuzumab in HER2-positive breast cancer. Dual inhibition of these pathways effectively reduces stemness markers and sensitizes resistant cells to HER2-targeted therapy. These findings lay the groundwork for multi-pathway combination strategies aimed at eliminating CSC populations, reducing EMT, and improving treatment outcomes in aggressive, therapy-resistant breast cancers.

## Data Availability

The data and materials generated and/or analyzed in this study are available from the corresponding author upon reasonable request.
